# Middle-distance running acutely influences the concentration and composition of serum bile acids: Potential implications for cancer risk?

**DOI:** 10.18632/oncotarget.17188

**Published:** 2017-04-18

**Authors:** Elisa Danese, Gian Luca Salvagno, Cantor Tarperi, Davide Negrini, Martina Montagnana, Luca Festa, Fabian Sanchis-Gomar, Federico Schena, Giuseppe Lippi

**Affiliations:** ^1^ Laboratory of Clinical Biochemistry, Department of Neurological, Biomedical and Movement Sciences, University of Verona, Verona, Italy; ^2^ School of Sport and Exercise Sciences, Department of Neurological, Biomedical and Movement Sciences, University of Verona, Verona, Italy; ^3^ Department of Physiology, Faculty of Medicine, University of Valencia and Fundación Investigación Hospital Clínico Universitario de Valencia, Instituto de Investigación INCLIVA, Valencia, Spain; ^4^ Leon H. Charney Division of Cardiology, New York University School of Medicine, New York, NY, USA

**Keywords:** bile acids, damage, exercise, sport, gastrointestinal cancer

## Abstract

**Background:**

This study was aimed to investigate the acute effect of medium-distance running on bile acids concentration and composition, in order to verify whether the positive impact of physical exercise on cancer risk may also be mediated by variation of bile acids concentration and composition in serum.

**Methods:**

The concentration and composition of serum bile acids was analyzed in 30 middle-aged and healthy recreational athletes with a reference liquid chromatography-mass spectrometry technique, immediately before and shortly after the end of the running trial. The concentration of bile acids after the run was adjusted for plasma volume change.

**Results:**

All athletes successfully completed the trial. After correction of values for the individual plasma volume change calculated after the run, the serum concentration of total bile acids was found to be significantly reduced by approximately 46%. A statistically significant decrease was observed for cholic, deoxycholic, chenodeoxycholic, ursodeoxycholic, glycoursodeoxycholic and hyodeoxycholic acids, whereas the concentration of the remaining compounds remained unvaried after the run. A considerable variation of bile acids profile was also observed. No significant association was found between running performance and variation of bile acids concentrations.

**Conclusion:**

These results show that middle distance running acutely decreases the concentration of total bile acids in serum, especially that of the more mutagenic and carcinogenic compounds, so providing an intriguing support to the favorable effects of physical exercise for lowering the risk of many gastrointestinal cancers.

## INTRODUCTION

Bile acid exposure has been convincingly identified as a potential risk factor for many forms of gastrointestinal (GI) cancers [1]. In fact, elevated secretion of bile acids is associated with an increased incidence of GI cancer [2]. It has been reported that high concentrations of secondary bile acids damage the GI epithelium as a result of DNA oxidative damage, inflammation, and activation of nuclear factor-kappa B (NFkB), playing thus an important role in GI cancer progression [3].

The bile acids are 24-carbon steroids produced from cholesterol in the liver. These compounds are usually divided into those directly synthesized by the liver (i.e., cholic acid [CA] and chenodeoxycholic acid [CDCA]), and those produced by bacterial metabolism of the two primary bile acids in the intestine (i.e., deoxycholic acid [DCA], lithocholic acid [LCA] and ursodeoxycholic acid [UDCA]. Additional metabolic pathways encompass the conjugation of CA with glycine or taurine to produce taurocholic acid (TCA) and glycocholic acid (GCA), along with the conjugation of CDCA with glycine or taurine to produce taurochenodeoxycholic acid (TCDCA) and glycochenodeoxycholic acid (GCDCA) [4]. Over 90% of the plasma or serum concentration of bile acids in humans is attributable to DCA, CA and their conjugates (i.e., TCA and GCA), as well as to CDCA and its conjugates (i.e., TCDCA and GCDCA) [4]. The most important biological functions of bile acids encompass the solubilisation of dietary lipids, fat-soluble vitamins and other nutrients, so that their intestinal adsorption can be substantially enhanced [5]. Notably, recent evidence showed that bile acids may exert pleotropic functions, such as regulation of glucose homeostasis, control of signalling events in the liver and overall regulation of energy expenditure [6].

The bile acid levels in faeces and blood may be influenced by a number of variables such as total energy intake or the amount and type of dietary fats and fibers [7]. Besides diet, additional factors may contribute to modify the synthesis and concentration of bile acids, so possibly including physical exercise. The potential interplay between physical activity and cancer is not insignificant, wherein the former exerts a variety of favorable health effects, including protective action against certain types of cancer [8]. In this regard, it has been reported that regular physical activity provides protection against GI cancer risk [9], although the mechanisms implicated remain to be understood.

Therefore, we designed this study to investigate the acute effect of medium-distance running (i.e., 21.1 km) on bile acids concentration and composition in the serum of 30 middle-aged and healthy recreational athletes, in order to verify whether the positive impact of exercise on GI cancer risk may also be mediated by a variation of bile acids concentration and composition in serum.

## RESULTS

The baseline concentration of hemoglobin, hematocrit and bile acids is shown in Table 1. The total concentration of bile acids pre-exercise was 3.20 μmol/L. GCDCA was the most represented bile acid, followed by CDCA, DCA, GDCA, GCA and CA. In agreement with published data [4], these 6 bile acids represented altogether 86% of the total concentration of bile acids in serum.

**Table 1:** Variation of bile acids after a 21 km, half-marathon run

Variable	Pre-exercise	Post-exercise	p
Body weight (kg)	73.7 (64.7-80.6)	73.6 (64.9-80.3)	0.308
Plasma volume change (%)	-	−1% (−4 to 4%)	-
Hemoglobin (g/L)	148 (139-153)	148 (137-154)	0.416
Hematocrit	0.45 (0.44-0.47)	0.45 (0.43-0.47)	0.332
Bile acids			
- CA (μmol/L)	0.16 (0.06-0.77)	0.06 (0.02-0.13)	0.004
- DCA (μmol/L)	0.32 (0.18-0.64)	0.10 (0.06-0.17)	<0.001
- CDCA (μmol/L)	0.39 (0.11-0.88)	0.12 (0.06-0.24)	0.001
- UDCA (μmol/L)	0.05 (0.01-0.15)	0.01 (0.00-0.02)	<0.001
- TCDCA (μmol/L)	0.09 (0.05-0.23)	0.14 (0.07-0.31)	0.068
- GCA (μmol/L)	0.20 (0.13-0.46)	0.15 (0.06-0.32)	0.397
- GDCA (μmol/L)	0.22 (0.16-0.29)	0.17 (0.10-0.33)	0.237
- GUDCA (μmol/L)	0.06 (0.04-0.08)	0.06 (0.04-0.06)	0.033
- GCDCA (μmol/L)	0.71 (0.51-1.39)	0.71 (0.47-1.15)	0.463
- HDCA (μmol/L)	0.14 (0.07-0.23)	0.07 (0.04-0.15)	0.003
- LCA (μmol/L)	0.01 (0.01-0.02)	0.01 (0.01-0.02)	0.132
- TCA (μmol/L)	0.02 (0.01-0.05)	0.02 (0.01-0.05)	0.189
- Total bile acids (μmol/L)	3.20 (1.76-6.16)	2.02 (1.20-3.10)	0.016

Results are shown as median and interquartile range (IQR).

CA, cholic acid; DCA, deoxycholic acid; CDCA, chenodeoxycholic acid; UDCA, ursodeoxycholic acid; TCDCA, taurochenodeoxycholic acid; GA, glycocholic acid; GCDCA, glycochenodeoxycholic acid; GUDCA, glycoursodeoxycholic acid; HDCA, hyodeoxycholic acid; GDCA, glycodeoxycholic acid; LCA, lithocholic acid; TCA, taurocholic acid.

All athletes successfully completed the 21.1 km trial, with a mean time of 1h52min42sec (range between 1h31min00sec and 2h31min11sec). The mean variation of hemoglobin, hematocrit and bile acids after the trial is shown in Table 1. No significant variation of both hemoglobin and hematocrit values could be detected after-exercise (Table 1), so that the plasma volume change was also insignificant (−1%; IQR, −4% to 4%). This is in agreement with the observation that the body weight did not significantly vary before and after the trial (p=0.308). After correction for individual plasma volume change, the concentration of total bile acids in serum was found to be significantly reduced, by approximately 46% (IQR, 15-60%), and a decreased concentration was observed in 23 out of 30 athletes (77%; p=0.030). As regards the specific bile acids, a statistically significant decrease was recorded for CA, DCA, CDCA, UDCA, GUDCA and HDCA, whereas the serum concentration of the remaining compounds was mostly unaffected. A trend toward increased values was noticed for TCDCA, though only approximating statistical significance. Interestingly, the number of athletes displaying a significant decrease of serum concentration of the single bile acid reached statistical significance for CA, DCA, CDCA, UDCA and HDCA.

**Figure 1:**
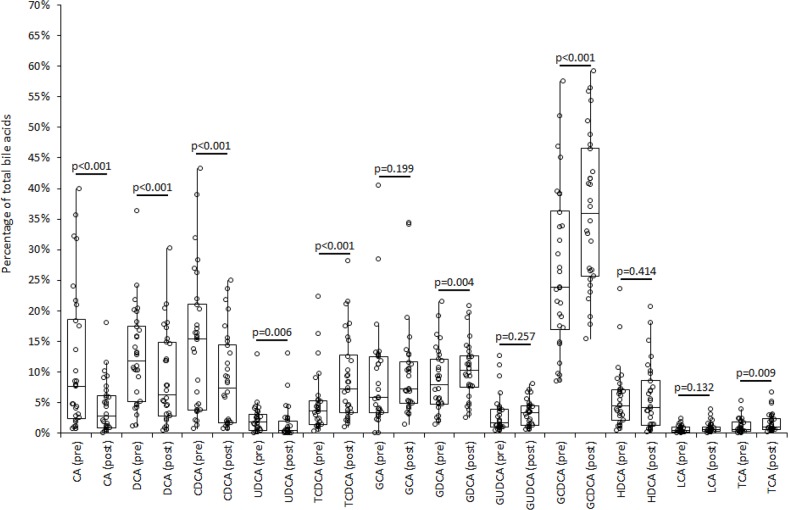
Composition of bile acids profile in serum immediately before (“pre”) and shortly after (“post”) middle distance running in 30 adult recreational athletes CA, cholic acid; DCA, deoxycholic acid; CDCA, chenodeoxycholic acid; UDCA, ursodeoxycholic acid; TCDCA, taurochenodeoxycholic acid; GA, glycocholic acid; GCDCA, glycochenodeoxycholic acid; GUDCA, glycoursodeoxycholic acid; HDCA, hyodeoxycholic acid; GDCA, glycodeoxycholic acid; LCA, lithocholic acid; TCA, taurocholic acid.

The variation of bile acids profile as percent of total bile acids is shown in Figure 1. A significant reduction of the percent value was hence observed for CA, DCA, CDCA and UDCA, a significant increase of percent values was observed for TCDCA, GDCA, GCDCA and TCA, whereas the percent value remained unvaried for GCA, GUDCA, HDCA and LCA. The number of athletes displaying a significant decrease of percent value reached statistical significance for CA, DCA, CDCA and UDCA, whereas a significant increase of percent value reached statistical significance for TCDCA and TCA (Table 2).

**Table 2:** Number of subjects with post-exercise decrease of bile acids concentration

Bile acids	Athletes with decrease of absolute serum concentration	p	Athletes with decrease of percent value over total bile acids concentration	p
CA	26/30 (87%)	0.003	24/30 (80%)	0.015
DCA	28/30 (93%)	<0.001	25/30 (83%)	0.007
CDCA	26/30 (87%)	0.003	25/30 (83%)	0.007
UDCA	28/30 (93%)	<0.001	25/30 (83%)	0.007
TCDCA	16/30 (53%)	0.398	1/30 (3%)	<0.001
GCA	20/30 (67%)	0.145	15/30 (50%)	0.500
GDCA	18/30 (60%)	0.302	9/30 (30%)	0.094
GUDCA	20/30 (67%)	0.145	12/30 (40%)	0.302
GCDCA	16/30 (53%)	0.398	8/30 (27%)	0.056
HDCA	25/30 (83%)	0.007	16/30 (53%)	0.398
LCA	19/30 (63%)	0.217	11/30 (37%)	0.217
TCA	13/30 (43%)	0.398	3/30 (10%)	0.001
Total bile acids	23/30 (77%)	0.030	-	

CA, cholic acid; DCA, deoxycholic acid; CDCA, chenodeoxycholic acid; UDCA, ursodeoxycholic acid; TCDCA, taurochenodeoxycholic acid; GA, glycocholic acid; GCDCA, glycochenodeoxycholic acid; GUDCA, glycoursodeoxycholic acid; HDCA, hyodeoxycholic acid; GDCA, glycodeoxycholic acid; LCA, lithocholic acid; TCA, taurocholic acid.

As predictable, a significant correlation was observed between running time and sex, whereas no significant associations were found between running performance and variation of bile acids concentrations, expressed as the ratio between pre- and post-exercise serum values. The associations between running time and variation of bile acids concentrations after the run remained non-significant also after including sex as a covariate in the correlation analysis

## DISCUSSION

Controversial information has been provided about the relationship between physical exercise and bile acids metabolism, mostly based on a limited number of cross-sectional studies. Sutherland et al carried out a small cross-sectional study including 14 male distance runners and 14 sedentary men [10]. In agreement with our findings in serum, the total fecal bile acid concentration was found to be significantly lower in athletes compared to the control population of sedentary men. Wertheim et al carried out a larger cross-sectional analysis of baseline fecal bile acid concentration in 735 participants of a phase III ursodeoxycholic acid chemoprevention trial [11]. Subjects in the highest quartile of adjusted hours per day of recreational activity displayed a 17% lower total fecal bile acid concentration compared to subjects in the lowest quartile, after adjustment for age, sex and body mass index. Opposite findings were instead reported in a subsequent study. Briefly, Prinz et al studied 43 female anorexic with broad activity patterns, and found that the total concentration of plasma bile acids was not associated with either metabolic equivalents/day (r=0.162; p=0.304) or total energy expenditure (r=0.147; p=0.352) [12]. This difference has been explained with the fact that bile acids in plasma were negatively correlated with the cognitive restraint of eating, so that their metabolism undergoes a compensatory adaptation to prevent further overeating after exercising.

Taken together, the results of our study demonstrate for the first time that exercise (but not the individual performance expressed as running time) acutely influences both the concentration and composition of bile acids in the blood of recreational athletes. Interestingly, middle-distance running was effective to acutely decrease the total concentration of serum bile acids, as well as the relative serum values of CA, DCA, CDCA, UDCA, GUDCA and HDCA, whereas the serum concentration of the remaining bile acids was mostly unaffected (Table 1). The composition of serum bile acids was also found to be substantially modified immediately after the run, since the relative contribution of some bile acids to the total amount was decreased (i.e., CA, DCA, CDCA and UDCA), whereas the relative contribution of other compounds was found to be increased (i.e., TCDCA, GDCA, GCDCA and TCA) (Figure 1).

These findings may have important implications for GI cancer biology. It has been clearly established that bile acids play an essential role in carcinogenesis, as supported by reliable epidemiological and biological evidence [13–16]. In particular, many studies found that an increased concentration of bile acids in both faeces and serum is strongly associated with the risk of developing many GI cancers, including cholangiocarcinoma, colorectal, pancreatic and gastric malignancies [1] [13–15]. The observation that also the serum concentration of these compounds may be associated with the risk of GI cancers is reasonable, since bile acids formed in the colon are then absorbed into the portal venous blood and then constantly released into systemic circulation [16]. Notably, the measurement of bile acids in serum or plasma has also some advantages over their assessment in stool. The biological matrix (i.e., the serum) can be obtained much more easily than the faeces, the measurements are more reliable and standardized, and recent dietary intake has a less important effect on bile concentration and composition of bile acids in serum than in stool.

As regards the biological link between bile acids and carcinogenesis, this association is supported by a variety of pro-carcinogenic effects that bile acids may exert, so including generation of reactive oxygen and nitrogen species, cell membrane perturbation, direct DNA damage, disruption of mitochondria, promotion of cell proliferation and mitotic events, reduced susceptibility to apoptosis, as well as activation of erbB2 and EGFR signaling cascades [13]. Therefore, our finding that the release of bile acids into the circulation is acutely reduced by approximately 46% immediately after exercise may bring an additional and reasonable explanation to the favorable impact of physical exercise for lowering the risk of GI cancers. Notably, some bile acids compounds (especially non-conjugated bile acids and DCA) were found to exert stronger pro-carcinogenic effects than others [17]. Accordingly, our observed decrease in the concentrations of serum CA (median reduction, 88%; IQR, 51-88%), CDCA (median reduction, 75%; IQR; 51-84%) and DCA (median reduction, 67%; IQR, 53-76%) after the run further reinforces this hypothesis. As regards the potential mechanisms linking exercise and variation of bile acids metabolism, it is well-established that endurance exercise acutely lowers serum cholesterol concentration, a finding that has also been previously confirmed in middle-distance runners [18]. The lower availability of cholesterol in blood during endurance exercise would hence represent a limiting step for the efficient synthesis of primary bile acids in the liver. As regards the relative variations of the different bile acids in serum, the substantial decrease of CA, DCA, CDCA and UDCA attests that the primary release into blood of non-conjugated bile acids and their main intestinal derivatives may be strongly impaired during exercise (Table 1), so contributing to decrease their relative contribution to the total serum pool of bile acids after the run (Figure 1). Conversely, the serum concentration of TCDCA, GDCA, GCDCA and TCA (i.e., products of conjugation of CDCA or CA with glycine or taurine) remained unchanged after distance running (Table 1), so explaining their increased contribution to the total serum pool of bile acids after the trial (Figure 1). Besides TCDCA, also GCDCA (the other product of CDCA conjugation) displayed a relatively increased value compared to the other bile acids, closely approaching statistical significance (Table 2). These two bile acids are those characterized by the higher bile acids uptake rate by human bile salt export pump (1400 pmol/mg per 30 s for TCDCA and 1000 pmol/mg per 30 s for GCDCA, respectively), which plays an important role in the clearance of bile acids [19]. Since the liver is subjected to exercise-induced cellular stress during prolonged exercise, it is hence conceivable that transitory hepatocyte sufferance may lead to a greater release of these two compounds into the circulation, so explaining the relative increase of their measurable values in plasma compared to the other bile acids.

In conclusion, our results confirm reliable evidence that exercise, particularly aerobic or endurance exercise (i.e., middle distance running), may acutely decrease the concentration of total bile acids, especially that of the more mutagenic and carcinogenic compounds. We provide thus an additional and putative explanation to the favorable effects exerted by physical exercise for lowering the risk of many GI cancers in which bile acids my act as promoters and/or contributors [20]. Unfortunately, we could not measure plasma bile acids at different time points after the run. Therefore, it remains to be established whether carcinogenesis can be abated by abruptly decreasing the concentrations of bile salts after endurance running and active lifestyle characterized by a lower volume of exercise would equally effective in reducing carcinogenesis.

## MATERIALS AND METHODS

This study was part of the “Run For Science,” a special event hold in Verona (Italy) in April 2014, and designed to study the physiological response of recreational athletes to half-marathon running (the full information of the event can be retrieved from the Verona University Website, at: http://www.dsnm.univr.it/?ent=iniziativa&id=5382, Last accessed, 12 February 2017). Specifically, 30 amateur runners (9 women and 21 men; mean age: 45 years, range 31-62 years; mean body mass index: 23.4, range: 19.8-27.9) successfully performed a 21.1 km (half-marathon) run. All athletes were part of an amateur running team, regularly engaged in recreational running (median training regimen, 219 min/week). The participants were asked to cease all exercise training and abstain from taking drugs or medications 48 hours before the event. No subject was regularly taking fibrates, bile acid sequestrants, niacin or statins. A control population was not included in this study since, as previously shown by Zheng et al, up to 2-4 hours fasting does not generate a significant variation of total bile acids values and bile acids composition in serum [21]. Therefore, all the changes potentially observed are almost entirely attributable to the effect of distance running.

The event developed as follows. The start was given at 9.30 AM and the full running distance (21.1 km) developed on a relatively flat route, located at 5 km from the town of Verona (35 m vertical gain, maximal slope of 1.8%). The weather conditions were optimal, i.e., partially sunny day with temperatures comprised between 12-19°C and humidity ranging between 55-75%. All participants run with a heart rate monitor (HRM) and were allowed to drink ad libitum during the run, but were asked to abstain from intaking food or caloric beverages, which were only allowed at the conclusion of the trial after blood sampling. Two blood collections were performed, the former just before the start of the run and the latter immediately after crossing the finishing line. The blood was collected in two primary blood tubes, the former without additives and the latter containing K_2_EDTA (Terumo Europe N.V., Leuven, Belgium). All samples were immediately conveyed by car and using a refrigerated transport box to the laboratory of clinical biochemistry and hematology of the University Hospital of Verona. Immediately after arrival to the laboratory, the blood tubes without additives were centrifuged for 10 min at 1300 x g, the serum was separated and then stored in aliquots at −70°C. A full blood count was instead immediately performed on K_2_EDTA blood tubes, using an ADVIA 2120 (Siemens Healthcare Diagnostics, Tarrytown, NY, USA).

The measurement of bile acids was performed as follows. A pre-exercise and post-exercise serum aliquot of each athlete was thawed at room temperature for 3 hours. The serum in the aliquots was mixed by 10-time gentle inversion and then measured with liquid chromatography-mass spectrometry (LC-MS). Separate measurements were performed in order to detect and quantify a panel of bile acids covering at least 99% of total bile acids contents in serum or plasma [21]: CA, DCA, CDCA, UDCA, TCDCA, GCA, GCDCA, glycoursodeoxycholic acid (GUDCA), hyodeoxycholic acid (HDCA), glycodeoxycholic acid (GDCA), LCA and TCA. Briefly, three deuterated internal standards (IS) were used for quantification: cholic-2,2,4,4-d4 Acid (CA-d4), chenodeoxycholic −2,2,4,4-d4 acid (CDCA-d4) and deoxycholic-2,2,4,4-d4 acid (DCA-d4). All reagents, so including UHPLC-grade ammonium formate, methanol and acetonitrile were obtained from Sigma Aldrich (St. Louis, MO, USA) or Santa Cruz (CA, USA). The separation was performed with a Nexera X2 series UHPLC (Shimadzu, Kyoto, Japan) equipped with a Phenomenex Kinetex C18 (500 × 2.1 mm, 2.6 μm) column. The temperature of the column was set at 50°C. The mobile phase A and B were 5 mM aqueous ammonium formate and acetonitrile and methanol (50:50), respectively. A gradient elution under 0.5 mL/min flow rate was carried out entailing 30% B for 0.3 min, linear increase to 90% B up to 5 min, followed by 90% B between 5-7 min and re-equilibration between 7-12 min with 30% B. The chromatographic system was coupled with a 4500 MD triple quadrupole MS detector (ABSciex, Darmstadt, Germany). The electrospray ionization was completed in negative mode, as follows: 30 psi (curtain gas), 550°C (Source Temperature) −3500 V (Ion Spray Voltage), 45 psi/55 psi (Ion Gas 1 and 2, respectively). Data were then recorded in multiple reaction monitoring mode (MRM), using nitrogen as collision gas. System operation, data acquisition and quantification were based on Analyst 1.6.2. software (AB Sciex) and Multiquant 3.0.2. (AB Sciex). The declustering potential, collision cell parameters and transitions were all optimized according to the various bile acids under to be measured.

A mixed stock solution of each bile acid standard was prepared in methanol and then diluted with methanol:water (50:50, v:v) to prepare a 6-point curve of calibration. The sample preparation was carried out in accord with published methods [22] [23] and minor modifications. Briefly, 500 μL of serum was spiked with 5μL of IS-mixture (24 μmol/L D4-CA, 25μmol/L D4-DCA, 25 μmol/L D4-CDCA), mixed with 1000 μL of acetonitrile and then vortexed for 1 min. After 15 min of centrifugation at 13000 x g, 500 μL of the supernatant were transferred to autosampler vials for UHPL-MS analysis. The injection volume was 2 μL. The limit of quantitation (LOQ), determined as a signal-to-noise ratio of ten, was below 20 nmol/L for all bile acid compounds. The intra-assay imprecision was comprised between 1.2-2.4%, whereas the inter-assay imprecision was comprised between 4.4-6.4%. The total concentration of bile acids in serum was obtained as the sum of data obtained from single measurement of each of the 12 bile acids. The statistical analysis was performed after correcting the absolute serum values of the single bile acids, as well as the total bile acid concentration, for the observed variation of plasma volume after exercise, using the validated formula of Dill and Costill [24].

Since the values of all variables did not follow a normal distribution, as established by Kolmogorov–Smirnov test, results were shown as median and interquartile range (IQR). The statistical analysis was also based on non-parametric tests, entailing Wilcoxon test for paired samples, chi square test with Yates’ correction and Pearson’s correlation, using Analyse-it (Analyse-it Software Ltd, Leeds, UK).

All subjects enrolled in this study provided a written consent. Likewise, this study was approved by the local ethical committee (Department of Neurological, Neuropsychological, Morphological and Movement Sciences, University of Verona, Italy) and was carried out in accordance with the Helsinki Declaration.
